# Valproic Acid-Like Compounds Enhance and Prolong the Radiotherapy Effect on Breast Cancer by Activating and Maintaining Anti-Tumor Immune Function

**DOI:** 10.3389/fimmu.2021.646384

**Published:** 2021-05-12

**Authors:** Zuchao Cai, David Lim, Guochao Liu, Chen Chen, Liya Jin, Wenhua Duan, Chenxia Ding, Qingjie Sun, Junxuan Peng, Chao Dong, Fengmei Zhang, Zhihui Feng

**Affiliations:** ^1^ Department of Occupational Health and Occupational Medicine, School of Public Health, Cheeloo College of Medicine, Shandong University, Jinan, China; ^2^ School of Health Sciences, Western Sydney University, Campbelltown, NSW, Australia; ^3^ College of Medicine and Public Health, Flinders University, Bedford Park, SA, Australia

**Keywords:** VPA-like compounds, radiotherapy, breast cancer, TAMs, M1-like macrophages, CD8^+^ T, vasculature

## Abstract

Inadequate sustained immune activation and tumor recurrence are major limitations of radiotherapy (RT), sustained and targeted activation of the tumor microenvironment can overcome this obstacle. Here, by two models of a primary rat breast cancer and cell co-culture, we demonstrated that valproic acid (VPA) and its derivative (HPTA) are effective immune activators for RT to inhibit tumor growth by inducing myeloid-derived macrophages and polarizing them toward the M1 phenotype, thus elevate the expression of cytokines such as IL-12, IL-6, IFN-γ and TNF-α during the early stage of the combination treatment. Meanwhile, activated CD8^+^ T cells increased, angiogenesis of tumors is inhibited, and the vasculature becomes sparse. Furthermore, it was suggested that VPA/HPTA can enhance the effects of RT *via* macrophage-mediated and macrophage-CD8^+^ T cell-mediated anti-tumor immunity. The combination of VPA/HPTA and RT treatment slowed the growth of tumors and prolong the anti-tumor effect by continuously maintaining the activated immune response. These are promising findings for the development of new effective, low-cost concurrent cancer therapy.

## Introduction

Breast cancer is one of the most common types of tumors in women, and radiotherapy (RT) is a mainstay of oncology treatment. In addition to the direct cytoreductive effect of RT in breast cancer, emerging evidence suggests that the generation of an anti-tumor immune response also plays an important role in the effectiveness of this treatment modality (1, 2).

A variety of different cell types within tumors have been described to undergo apoptosis after local irradiation, these include T cells, stromal cells, and vascular endothelial cells, which limited the therapeutic effect to some extent and increased the possibility of immune escape (2). At the same time, RT paradoxically promotes metastasis and invasion of cancer cells by inducing the epithelial–mesenchymal transition (EMT), and can even cause tumor recurrence (3), which are the main obstacles to the successful treatment of cancer, and remains the important cause of mortality in patients receiving RT (4). New therapeutic strategies, such as combining immunotherapy with RT are being trialed (5).

Breast cancer has a complex microenvironment consisting of malignant cells, resident histiocytes such as adipocytes and recruited cell types, which play an important role in the progression of breast cancer to malignancy and resistance to treatments (6). Among them, macrophages play a pivotal role. Tumor-associated macrophages (TAMs), one of the main types of immunosuppressive cells in the tumor microenvironment, are key players in tumor immune escape, a major obstacle to cancer immunotherapy (7, 8). In the overwhelming majority of tumors, TAMs stimulate tumor cell migration, invasion, intravasation as well as the angiogenic response required for tumor growth (9–11). Clinicopathological studies have suggested that TAMs accumulation in tumors is correlated with a poorer clinical outcome (12). In human breast carcinomas, high TAMs density is correlated with poorer prognosis (13). Depending on the microenvironmental presence, macrophages are polarized into two distinct phenotypes, the classically activated (M1) or the alternative activated (M2) macrophages. TAMs closely resemble the M2-polarized phenotype (14). Recent studies have shown that polarizing TAMs toward M1 phenotype can effectively treat tumors (15–19). This suggests that macrophages have plasticity, which can restore the anti-tumor properties of TAMs for the treatment of tumors (20). Therefore, TAMs are considered as one of the important therapeutic targets to improve the efficacy of immunotherapy, and the search for novel drugs that can modulate the TAMs phenotype holds promise for safer and more effective oncology treatment.

On the other hand, activation and recruitment of cytotoxic lymphocytes (CTLs) have been recognized as key to effective immunotherapy for solid tumors. Among them, CD8^+^ T cells are essential to inhibit the occurrence and development of solid tumors, because once these cells exert full cytotoxicity, they can eliminate tumor cells (21). Most solid tumors include a variety of immune cells, such as regulatory T cells and TAMs, which can inhibit CTLs function (22, 23). It was reported that the depletion of TAMs enhances CD8^+^ T cell-mediated anti-tumor immunity in a mouse model of breast cancer (24). Therefore, therapies targeting the immune system hold great promise for the treatment of cancer (25, 26).

In recent years, some scholars have reported that a histone deacetylase inhibitor (HDACi), TMP195 can switch the major macrophage type in tumors from TAMs to the high phagocytic macrophages in mice mammary tumors (27). In this model, TMP195 activates immune pathways, and synergistic anti-PD1 antibodies and chemotherapy significantly inhibited tumor development. This HDACi, which has a stable and effective regulatory effect on the immune system, hold great potential as it targets specifically immune cells, resistance to treatment is rare as compared with those agents which directly act on the tumor cells (28, 29). The other HDACi, valproic acid (VPA), a well-tolerated anti-epileptic agent used since the 1970s, has also received attention recently as a possible concurrent therapy to RT. Many researchers have demonstrated that VPA-like compounds can kill a variety of tumor cells, including glioma (30), breast cancer (31), prostate cancer (32), while sensitizing tumor cells to RT or chemotherapy through its effect on DNA repair (33–35). It was not clear whether VPA and VPA-like compounds reported sensitization of tumor cells to RT or chemotherapy was associated with the regulation of immune function.

Therefore, in our study, we used a well-established animal model of breast cancer that does not affect tumor immune function (36) to explore whether VPA and VPA-like compounds may also have the ability to activate immune pathways, and when co-administered with RT can better inhibit the development of tumors.

## Methods and Materials

### Establishment of a Breast Cancer Model

Detailed steps are reported in our previous article (35). In brief, female Sprague–Dawley (SD) rats were purchased from Peng Yue Laboratory Animal Co. Ltd., Jinan, China. The studies of animal tissue were performed in accordance with the requirements of the Shandong University Human and Animal Ethics Research Committee (project identification code 81472800, approved 3 March 2014). A single dose of 1 ml 7,12-dimethylbenz[α]anthracene (DMBA) oil was administered to 50-day-old SD rats through intragastric gavage (37, 38). At 40–60 days after gavage, primary tumors could be detected through palpation around the breast. The tumor size, location and appearance were recorded weekly and measured with Vernier Caliper. Tumor volume was calculated according to the clinical standard formula “Volume (V; mm^3^) = Length (L) ∗ Width (W)^2^ ∗ 0.5”.

### Drug Treatment and Radiotherapy in Rats

The tumor-bearing rats were given an intraperitoneal injection of saline, VPA (BP452, Sigma) or HPTA (H0964, TCI) twice a day for 6 consecutive days. RT was applied to rats by using X-ray Irradiator (X-RAD225 OptiMAX, Pxi) as shown in **Figure S1**. Four fractionated doses of 2 Gy were utilized in our study. The specific methods are as follows: When irradiating, we fixed the rat and placed it on the round plate. And the hollow cylinder indicated by the red arrow is used for the precise irradiation of the tumor. The inside diameter of the hollow cylinder is 2 cm, and the tumor was exposed to radiation here (as indicated by the red arrow). And the X-ray aperture was selected to match the diameter of the tumor. The cylinder is made of solid copper, allowing full protection of the rest of the body.

### BrdU Incorporation and HE Staining

5-Bromo-2′-deoxyuridine (BrdU) (B5002, Sigma) was injected intraperitoneally at a dose of 100 mg/kg 24 h before tissue harvest. Tumor tissues and normal breast were fixed overnight in 4% paraformaldehyde solution, embedded in paraffin and serially sectioned 5 μm thick for hematoxylin and eosin (HE) staining according to the manufacture’s procedures guideline.

### Immunohistochemistry (IHC)

The avidin–biotin immunoperoxidase method was used for deparaffinized zinc formalin-fixed, paraffin-embedded sections. Specific methods are detailed in our previous article (27). The primary antibodies including CD11b (1:5,000, ab133357, Abcam), F4/80 (1:200, 123101, BioLegend), CD68 (1:500, GB11067, Servicebio), Cleaved caspase-3 (1:300, 9661, Cell Signaling), BrdU (1:50, B44, BD), Ki67 (1:400, 12202, Cell Signaling), CD8 (1:500, GB11068, Servicebio), granzyme-B (1:200, sc-8002, Santa Cruz), followed by incubation with secondary antibodies: biotinylated goat anti-mouse IgG (1:300, BA-9200, Vector), biotinylated goat anti-rat IgG (1:300, BA-9400, Vector) and biotinylated goat anti-rabbit IgG (1:300, BA-1000, Vector). Images were taken through a light microscope (Olympus).

### Immunofluorescence

Specific methods are detailed elsewhere (33, 39). The primary antibodies including CD11b (1:1,000, ab133357, Abcam), F4/80 (1:200, 123101, BioLegend), EpCAM (1:200, sc-66020, Santa Cruz), CD31 (1:200, GB12063, Servicebio), followed by staining with Alexa Fluor® 594 goat anti-mouse IgG(H+L) (1:300; A11032, Molecular probes), Alexa Fluor® 488 chicken anti-rabbit IgG(H+L) (1:300; A21441, Molecular probes). Images were taken using Zeiss 880 Confocal Microscope and analyzed on Leica Microsystems imaging software. Composite images and pseudo-colored images were generated using Fiji software and images were captured using a laser confocal microscope.

### Real-Time Quantitative Reverse Transcriptase PCR (qRT-PCR)

The tumors were rapidly extracted after the tissues were harvested, snap-frozen in liquid nitrogen, and stored at −80°C before being used for qRT-PCR analysis. The RNA was extracted from whole tumor tissue according to the RNA prep Pure Tissue Kit (Tiangen) protocol. For the cellular experiment, we extracted RNA according to the FastPure Cell/Tissue Total RNA Isolation Kit (vazyme) and the isolated RNA was quantified by NanoDrop ND-2000 spectrophotometer (Nadro Drop Technologies, Wilmington, DE, USA). cDNA synthesis was performed using the ReverAid First Strand cDNA Synthesis Kit (Thermo). Finally, specific primers and Maxima SYBR Green (Thermo) were used, and qRT-PCR analysis was performed on Light Cycler^®^ 480II (Roche Applied Science, Indianapolis, IN, USA) using 1 μL of each primer and 1 μL of cDNA. The levels of the relative genes and the internal reference gene (GAPDH) expressed were measured, and the C_t_ values (threshold cycle number) of the target gene and the reference gene were calculated according to the Light Cycler^®^ 480 Software release 1.5.0 SP4 software, use 2^−ΔΔ^ C_t_ method. Sample from the DMBA-induced breast cancer was used as control sample, and the expression of the target gene of each group was compared. ΔΔC_t_ = experimental group ΔC_t_ − control group ΔC_t_, ΔC_t_ = (average C_t_ of the target gene of the control sample - average C_t_ of the control sample GAPDH) (40, 41). The primer sequences used in this study are listed in **Table S1**.

### Cell Culture

MCF7 and RAW264.7 cell lines were purchased from American Type Culture Collection (ATCC) and maintained in DMEM (12100046, Gibco) medium with 10% Fetal Bovine Serum (10270106, Gibco) and 1% Penicillin-Streptomycin (V900929, Sigma). All cells were confirmed to be mycoplasma-free, and maintained at 37°C and 5% CO_2_.

### Cytokine Detection in Macrophage Lysate

MCF7 cells were seeded in P60 dishes followed by 500μM VPA, 15μM HPTA and 100 ng/ml LPS (L8880, Solarbio) treatment for 24 h. The culture was centrifuged to collect the medium supernatant, which is subsequently added to the P35 dishes seeded with RAW264.7 cells. After 24hrs, RAW264.7 cells were lysed by repeated freeze-thawing in PBS, and lysates were collected. Cytokines detection (IL-12, IL-10, TNF-α, IFN-γ) were performed using ELISA kits (1211232, 1211002, 1217202, 1210002, DAKEWE, China).

### Primary Culture and Stimulation of Human Peripheral Blood Mononuclear Cells (PBMCs)

Whole blood samples were collected from healthy donors after obtaining informed consent in accordance with the National Regulations on the Administration of Human Genetic Resources, China. The ethics for this part of the study was approved by the Shandong University Human and Animal Ethics Research Committee’s requirements (project identification code 81472800, approved on 3 March 2014). PBMCs were isolated from whole blood using Lymphocyte Isolate (LTS1007-1, TBDScience, China) density gradient centrifugation. The PBMCs were maintained in RPMI 1640 (12633012, Gibco) medium with 10% Fetal Bovine Serum (10270106, Gibco) and 1% Penicillin–Streptomycin (V900929, Sigma).

PBMCs were isolated, and cells were seeded at 5 × 10^5^ in 24-well plate coated with CD3 (5 μg/ml) (B287689, BioLegend) at 4°C overnight, 500 μl/well, and added to CD28 (1 μg/ml) (B281555, BioLegend) with IL-2 (10 ng/ml) (031612, PEPROTECH) and maintained at 37°C and 5% CO_2_ for 3 days. Subsequent experiments were performed after sufficient cells were reached.

### Flow Cytometry

Lysates were extracted following the macrophage factor detection step and added to the already activated PBMC cells at a 1:3 of medium volume ratio for 5 days in culture. Then PBMCs were collected, washed three times with PBS, incubated with the CD3 (B278047, BioLegend), CD4 (B310677, BioLegend) and CD8 (B311544, BioLegend), and centrifuged to collect cells. Cells were washed three more times with PBS and resuspended as a single cell suspension for flow cytometry.

### Co-Culture of Tumor Cells With PBMCs

Lysates were extracted following the macrophage factor detection step and added to the already activated PBMC cells at a 1:3 of medium volume ratio for 5 days in culture. 4 × 10^5^ MCF7 cells were seeded on the lower chamber of the transwell (Corning #3412, 24 mm Transwell^®^ with 0.4 µm Pore Polycarbonate Membrane Insert), and 2 Gy irradiation treatment was administered after the cells had fully adherent growth. At the end of irradiation, 2 × 10^5^ PBMC cells cultured for 5 days were transferred to the upper chamber of the transwell for co-cultured for 24 h. The tumor cells in the lower chamber were subjected to MTT to detect the number of viable cells.

### MTT

MCF7 cells were seeded in lower chamber of a 6-well transwell at a density of 4 × 10^5^ cells per well. Following treatments, MTT solution (5 mg/ml, Sigma) was added to the treated cells and incubated for 4 h at 37°C. Then the medium was replaced with dimethyl sulfoxide. After mixing, 120 μl was added to each well in a 96-well plate. The absorbance of the solution was measured using an enzyme immunoassay analyzer at 540 nm.

To determinate the effect of IR on the growth of macrophage, RAW264.7 were seeded at a density of 2 × 10^3^ cells per well in 96-well plate, and treated with 4 and 8 Gy after the cells had attached, and the growth of the cells observed by MTT assay after 72 h.

### Statistical Analysis

All statistical analyses were performed with Student’s t-test on SPSS Statistics for Windows, version 23.0 (Armonk, NY: IBM Corp; licensed to Shandong University) and represented as mean ± SD. The *P* values were designated as: *, *P <*0.05; **, *P <*0.01, indicating a statistically significant difference.

## Results

### VPA/HPTA Enhanced Radiotherapy Effect to Inhibit Tumor Growth in Rats With Breast Cancer

**Figure 1:**
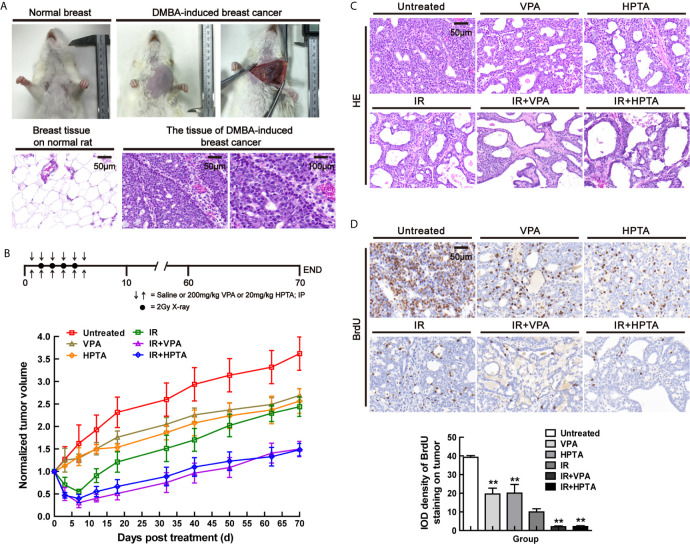
VPA/HPTA enhanced radiotherapy effect to inhibit tumor growth in rats with breast cancer **(A)** Normal breast and DMBA-induced breast cancer of rats under gross observation. HE staining for the morphology of normal tissue and DMBA-induced breast cancer. **(B)** The tumor-bearing rats were given intraperitoneal injection of saline, 200 mg/kg of VPA or 20 mg/kg of HPTA twice a day for 6 consecutive days in combination with 2Gy of radiotherapy once a day for 4 consecutive days. The change in tumor volumes in different groups after treatment, which was normalized by untreated group. **(C)** HE staining for the morphology of tumors in different groups. **(D)** IHC was performed on tumor sections with BrdU, a marker of proliferation. Quantitation as a percentage of total tissue is shown to the right of representative images. Each data point in the graphs was from three independent experiments (mean ± SD). *P*-values were calculated by Student’s t-test (*P < 0.05, **P < 0.01).

To study whether VPA/HPTA can enhance the effect of radiotherapy *in vivo*, we used the primary breast cancer model in rats induced by the environmental carcinogen DMBA, which was previously described and employed in related studies (33, 35, 36). In brief, around 40 days after DMBA gavage to female SD rats, lumps in the breast sites were found. The shape of lumps in the location of mammary glands was irregular (**Figure 1A**). By HE staining, when compared with the normal breast tissue, a monotonous population of cells, poorly circumscribed, infiltrating the surrounding soft and adipose tissues, cords and nodules of atypical epithelial cells, with some duct or gland formation, indicating that breast cancer in rats was successfully induced. Next, the dose of VPA/HPTA and radiotherapy were determined for the tumor treatment in this animal model. Reported studies of VPA on glioblastoma utilized intraperitoneal injection of VPA in the range from 150 to 600 mg/kg (42), here, we choose 200 mg/kg as the treatment dose of VPA, which was the same as that used to treat the cells (0.5 mM) in our working system (33, 35). 20 mg/kg HPTA was adopted as this is closest to the 200 mg/kg VPA previously utilized in cell culture (0.015 mM) (34). Four fractionated doses of 2 Gy, based on previous studies, were utilized (43, 44). The workflow of our experimental design is detailed in **Figure 1B** upper.

During the early observation, the growth of tumors in VPA/HPTA-treated rats was inhibited (*P <*0.05). Compared with the RT-alone group, the reduction of breast cancer volume in the VPA/HTPA treatment groups was significantly more (*P <*0.01). On the 10th day post-treatment, the morphological structure of tumors was observed by HE staining (**Figure 1C**). The VPA/HPTA treatment led to vacuole structures formation in the breast cancer tissue as compared with the untreated control group; there were more vacuoles structures and number of necrotic cells after the RT, and larger necrotic areas and cells were seen in the tissues in the combination treatment groups. The morphological results are consistent with the above findings. The results demonstrated that 200 mg/kg VPA or 20 mg/kg HPTA can effectively enhance RT for breast cancer in our rat working model.

We next tested the cell proliferation ability in the tumor using both BrdU and Ki67 markers. BrdU IHC staining results showed that VPA/HPTA treatment significantly reduced the proliferation of tumor cells, the reduction was significantly greater in the combination treatment groups (*P <*0.01, **Figure 1D**). Similar results were noted with the Ki67 proliferation marker (*P <*0.01, **Figure S1A**). The IHC findings were consistent with the gross observation and measurement. In conclusion, we highlight that the combination of both treatment modalities is superior to each treatment modality alone.

### VPA/HPTA Activates the Macrophages and Reprograms TAMs Polarization Towards M1 Phenotype in Irradiated Breast Tumor at the Early Stageof the Treatment

Other scholars had reported that TMP195 has a macrophage-mediated immune effect (27), so we next studied the macrophages in the tumor microenvironment to investigate whether VPA and VPA-like compound (HPTA) may have a similar effect during RT treatment in our working model.

**Figure 2:**
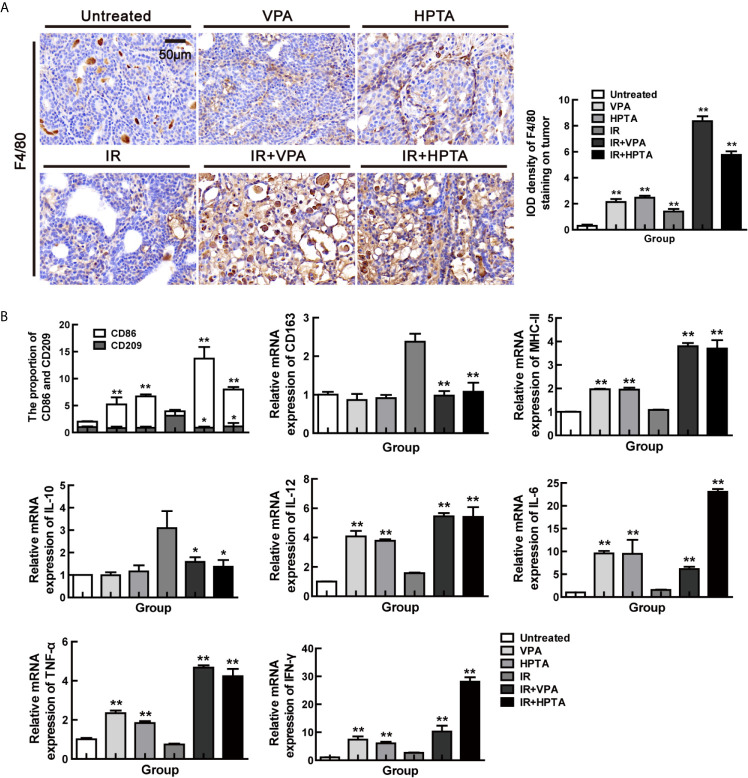
VPA/HPTA activates the macrophages and reprograms TAMs polarization towards M1 phenotype in irradiated breast tumor at the early stage of the treatment **(A)** IHC was performed on tumor sections with the macrophage-specific marker F4/80 to assess infiltration of macrophages, and representative quantitation and images are shown. **(B)** The mRNA expression levels of CD86, CD209, CD163, MHC-II, IL-10, TNF-α, IFN-γ, IL-6 and IL-12 in DMBA-induced breast tumors in rats were determined by real-time PCR. Data were normalized to untreated group. Each data point in the graphs was from three independent experiments (mean ± SD). *P*-values were calculated by Student’s t-test (*P < 0.05, **P < 0.01).

We used the macrophage marker F4/80 for IHC staining (**Figure 2A**) and found that after VPA/HPTA treatment, the macrophages increased significantly in the tumor, while in the combination treatment groups, there were a further substantial increased (*P <*0.01). Similar results were observed for the other macrophage marker, CD68 (*P <*0.01, **Figure S2**). The data indicate that the immune system is activated by VPA/HPTA in response to RT.

TAMs, being a M2 macrophage, play a key role in cancer immune escape. We next investigate whether VPA/HPTA treatment may be able to switch the polarization of macrophages to the pro-inflammatory M1 phenotype. As shown in **Figure 2B**, VPA/HPTA treatment alone significantly promoted an increase in the cell population expressing M1 marker (CD86; *P <*0.01) and M1 function markers (IL-12, IL-6, MHC-II, IFN-γ and TNF-α; *P <*0.01) at the transcriptional level in the tumor. The M2 macrophage marker (CD209 and CD163) and the function marker (IL-10) also had no significant change. For the RT-alone group, M2 macrophages, but not M1 macrophages, were significantly increased compared with the untreated control group. Meanwhile, in the combination treatment groups, the increase in M1 marker and function markers and decrease in M2 marker (CD209 and CD163) and function marker (IL-10) was further amplified (*P <*0.01). The data suggest that VPA/HPTA can reverse and further activate the RT-induced immune pathway at the early stage after the RT treatment.

### VPA/HPTA Regulates Myeloid-Derived Macrophages to Enhance Radiotherapy Effect in Breast Cancer at the Early Stage of Treatment *In Vivo*

**Figure 3:**
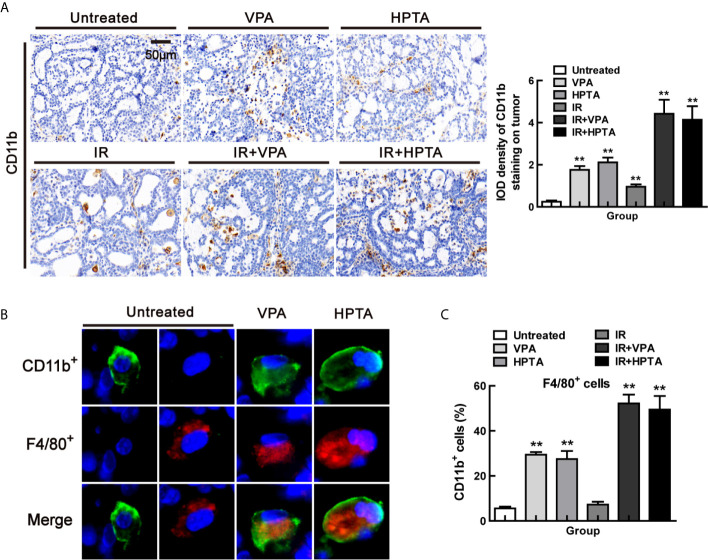
VPA/HPTA regulates myeloid-derived macrophages to enhance radiotherapy effect in breast cancer at the early stage of treatment *in vivo* **(A)** IHC was performed on tumor sections with the myeloid marker CD11b to assess infiltration, representative quantitation and images are shown. Immunofluorescence co-staining of myeloid derived cells (CD11b+: green) and macrophages (F4/80+: red), and representative images **(B)** and quantitation **(C)** are shown. Each data point in the graphs was from three independent experiments (mean ± SD). *P*-values were calculated by Student’s t-test (*P < 0.05, **P < 0.01).

We next explore the origin of the macrophages which were recruited into the tumor microenvironment by VPA/HPTA. Some scholars have reported that CD11b, a marker of myeloid-derived differentiated cells, can promote bone marrow cells to develop into macrophages and then inhibit tumor growth (45). Therefore, we performed IHC analysis of tumor tissues in each group with CD11b, the results showed that CD11b^+^ cells were significantly increased after VPA/HPTA treatment (1.76 ± 0.24/2.11 ± 0.31) (*P <*0.01, **Figure 3A**), there was a small increase after RT-alone (0.95 ± 0.15, *P <*0.05). We noticed a substantial increase in CD11b^+^ cells with the combination treatment (4.42 ± 0.94/4.14 ± 0.91) (*P <*0.01). The data demonstrate that VPA/HPTA can induce an increase in CD11b^+^ cells in the tumor.

Next, to verify the source of VPA/HPTA-induced macrophages, we employed co-localization staining of CD11b and F4/80 markers. As shown in **Figure 3B**, all F4/80^+^ cells co-localized with CD11b, and the proportion of the cells (CD11b^+^, F4/80^+^) increased significantly after VPA/HPTA treatment (29.4%/27.5%) (*P <*0.01, **Figures 3C** and **S3A**). This proportion was further increased in the combination treatment groups (52.2%/49.4%) (*P <*0.01), but not in the RT-alone group (*P >*0.05). The data demonstrate that the increased macrophages in the tumor may be of myeloid origin, which can be recruited into the tumor microenvironment by VPA/HPTA.

To distinguish whether the increased macrophage population were the resident macrophages in the tumor, the ability of RAW264.7 macrophages was tested after IR and VPA/HPTA combination treatment by MTT assay *in vitro*. We found that the ability of the macrophages irradiated with 4 and 8Gy was significantly decreased (**Figure S3B**, *P <*0.01); however, VPA/HPTA treatment did not cause a further decrease in the cell ability (*P >*0.05). We concluded that the previously observed increased macrophage population is likely from non-tumor resident macrophages, the myeloid-derived macrophages may be recruited from other tissues.

### VPA/HPTA-Activated Macrophages Are Highly Phagocytic in Breast Tumors at the Early Stage of Treatment *In Vivo*

**Figure 4:**
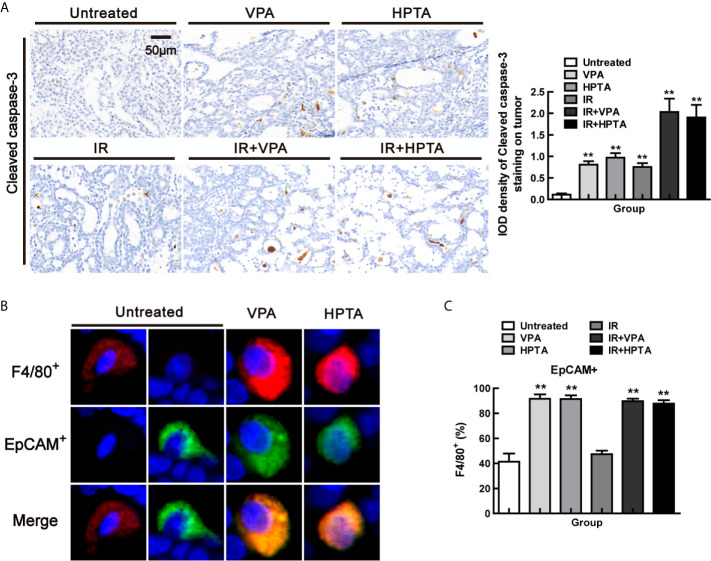
VPA/HPTA-activated macrophages are highly phagocytic in breast tumors at the early stage of treatment *in vivo* **(A)** IHC was performed using the cleaved caspase-3 to identify apoptotic bodies within macrophages, representative images and quantitation are shown. Phagocytosis of breast tumor cells was quantified as the proportion of F4/80+ macrophages (red) that contain intracellular EpCAM+ (green), a marker of breast tumor cells by immunofluorescence, representative images **(B)** and quantitation **(C)** are shown. Data were normalized to untreated group. Each data point in the graphs was from three independent experiments (mean ± SD). *P*-values were calculated by Student’s t-test (*P < 0.05, **P < 0.01).

To determine the effect of VPA/HPTA-activated macrophages on tumors, we found that the proportion of apoptotic cells (Cleaved caspase-3^+^) was increased after VPA/HPTA treatment and RT-alone treatment, this was further increased after the combined treatment (2.03 ± 0.43/1.90 ± 0.41, *P <*0.01, **Figure 4A**), suggesting that the combination treatment promoted the apoptosis of tumor cells.

Phagocytosis of breast tumor cells was quantified as the proportion of F4/80^+^ macrophages that contain intracellular EpCAM, a marker of breast tumor cells. By co-localization staining with F4/80 and EpCAM markers (**Figure 4B**), we found that the proportion was increased significantly both in the VPA/HPTA-alone and the combination treatment groups (89.61%/87.73%) (*P <*0.01, **Figures 4C** and **S4**). Thus, the macrophages induced by VPA/HPTA are highly phagocytic, which we concluded is helpful to enhance the RT effect in eliminating tumor cells.

### VPA/HPTA Reinforces the Anti-Tumor Effect of Radiotherapy by Activating CD8^+^ T Cell-Dependent Anti-Tumor Response and Inducing Vascular Normalization *In Vivo* at the Early Stage of the Treatment

**Figure 5:**
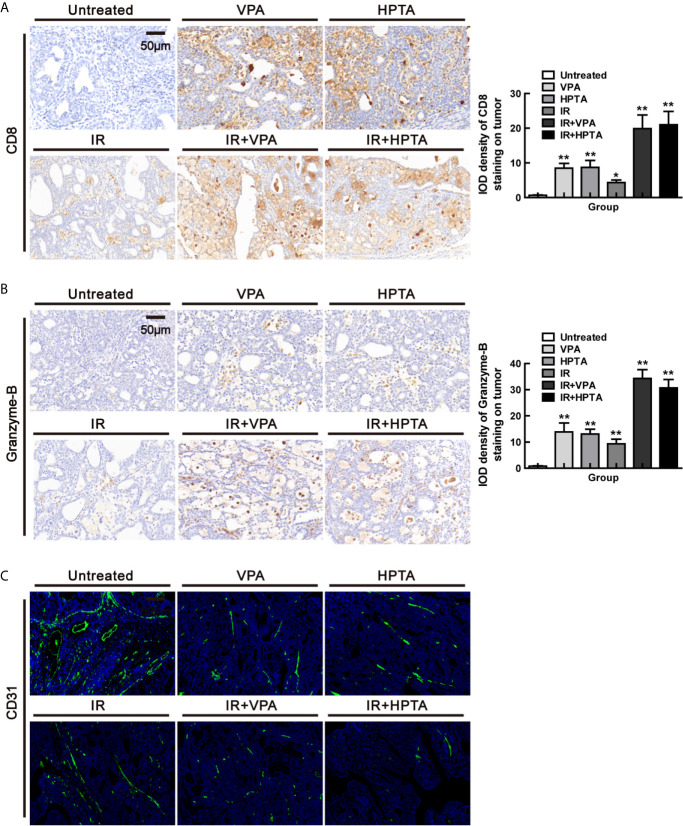
VPA/HPTA reinforces the anti-tumor effect of radiotherapy by activating CD8+ T cell-dependent anti-tumor response and inducing vascular normalization in vivo at an early stage of the treatment. **(A)** IHC was performed on tumor sections for the marker CD8. Quantitation as a percentage of total tissue is shown to the right of representative images. **(B)** IHC was performed on tumor sections with the marker granzyme-B. Quantitation as a percentage of total tissue is shown to the right of representative images. **(C)** Immunofluorescence staining of tumor vessels (CD31+: green) and representative images are shown. Each data point in the graphs was from three independent experiments (mean ± SD). *P*-values were calculated by Student’s t-test (*P < 0.05, **P < 0.01).

TAMs can target CD8^+^ T cells and inhibit immune rejection of tumor cells through various mechanisms (46), while IL-12 secreted by M1 cells can activate CD8^+^ T cells to stimulate an anti-tumor response in solid tumor models (47, 48). CD8^+^ T cells mediate the most important anti-tumor immune response *in vivo*, and most cancer immunotherapy approaches aim to evoke, promote and enhance the specific anti-tumor activity of CD8^+^ T cells (49). Since we found that VPA/HPTA promoted pro-inflammatory M1 phenotype and increased IL-12 expression in our study, we next examined whether VPA/HPTA can activate CD8^+^ T cells to be involved in the anti-tumor response. The results in **Figure 5A** showed that VPA/HPTA treatment induced an increase in CD8^+^ T cells population (*P <*0.01), which was also modestly increased in the RT-alone treatment group (*P <*0.05). The combination treatment further significantly increased the CD8^+^ T cells population (19.85 ± 5.61/20.00 ± 5.43) (*P <*0.01). Granzyme-B, the functional marker of CD8^+^ T cells, was also increased in the combination treatment groups (**Figure 5B**), indicating that VPA/HPTA activated the CD8^+^ T cells and thus enhanced the RT effect in the tumor, which may be associated with IL-12 secreted by anti-tumor M1-type macrophages.

The tumor-promoting TAMs contribute to abnormalities in tumor vasculature (9, 50–52), while anti-tumor M1 macrophages are associated with anti-angiogenic effects including vascular pruning and normalization (53). Studies have shown that IFN-γ can interfere with the integrity of blood vessels and affect the progression of tumors (27, 39). Since we discovered that VPA/HPTA increased mRNA level of IFN-γ of M1 function markers in our study, we next examine whether VPA/HPTA can influence angiogenesis in the tumor. The results of CD31, the markers of endothelial blood vessel, demonstrated that VPA/HPTA treatment, as well as the RT-alone treatment, reduced the size, density and aberrantly branches of the vasculature, and the effect was augmented in the combination treatment groups (**Figure 5C**). These findings suggest that VPA/HPTA combined with radiotherapy can inhibit tumor neovascularization, such action is associated with IFN-γ secreted by anti-tumor M1 macrophages exhibiting anti-angiogenic properties.

### VPA/HPTA Prolong the Radiotherapy Effect of Breast Cancer *via* Maintaining the Durability of Anti-Tumor Immune Response *In Vivo*

Our results on the tumor growth revealed an interesting phenomenon. As shown in **Figure 1B**, the tumor volume significantly decreased in the first week after the RT-alone treatment, and then started to increase after that. At the end of the observation period (70 days), the tumor volume elevated to about 2.5 times than that before RT treatment. Surprisingly, for the combination treatment groups, the tumor volume grew slowly after an initial decrease in the first week, and subsequently the tumor volume was increased about 0.5 times than that before the treatment at the end of the observation period, indicating that both VPA and HPTA could significantly prolong the RT effect in inhibiting tumor growth. We speculated that this effect may be associated with anti-tumor immune response activated at the early stage of the treatment, so we further analyzed the immune state in the tumor at 70 days after treatment.

**Figure 6:**
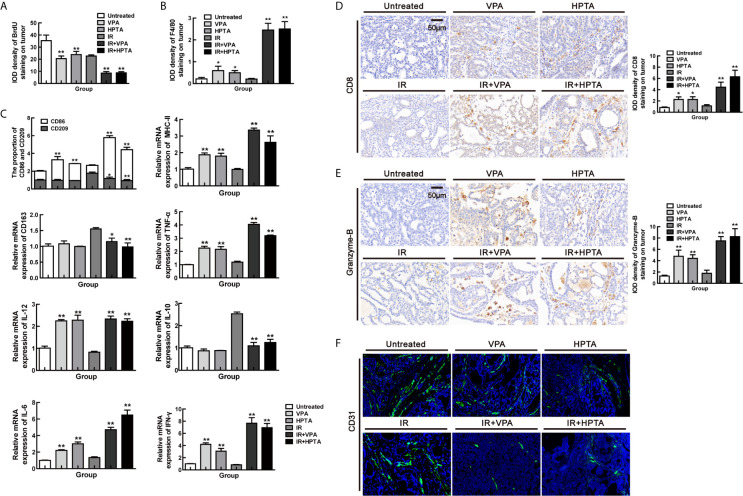
VPA/HPTA prolong the radiotherapy effect of breast cancer via maintaining the durability of anti-tumor immune response *in vivo* Tumor tissues were analyzed 70 days after treatment. Quantitative analysis of BrdU **(A)** and F4/80 **(B)** immunohistochemistry. **(C)** The mRNA expression levels of CD86, CD209, CD163, MHC-II, IL-10, TNF-α, IFN-γ, IL-6 and IL-12 in DMBA-induced breast tumors in rats were determined by real-time PCR. Data were normalized to the untreated group. **(D)** IHC was performed on tumor sections for the marker CD8. Quantitation as a percentage of total tissue is shown to the right of representative images. **(E)** IHC was performed on tumor sections for the marker granzyme-B. Quantitation as a percentage of total tissue is shown to the right of representative images. **(F)** Immunofluorescence staining of tumor vessels (CD31+: green) and representative images are shown. Each data point in the graphs was from three independent experiments (mean ± SD). *P*-values were calculated by Student’s t-test (*P < 0.05, **P < 0.01).

Firstly, HE staining showed that there were still large necrotic areas and cells in the combination treatment groups (**Figure S5A**). The results of BrdU showed that the cells in the untreated group were still high-proliferative, the proliferative capacity in the RT-alone group was the same as in the VPA/HPTA-alone groups, which was consistent with the tumor growth (**Figure 1B**), but was lower than that in the untreated control group (*P <*0.01). While, the combination treatment groups still showed much lower proliferative capacity (*P <*0.01, **Figures 5B** and **6A**). Similar findings were noted with Ki67 (*P <*0.01, **Figure S5C**). The data indicate that the tumor growth was inhibited in the combination treatment at the later stage of RT treatment.

Subsequently, F4/80 IHC results suggested that macrophages were still active in the combination treatment group (*P <*0.01, **Figures 6B** and **S5D**), although not as evident as in the early stage, as can be seen largely by the CD68 staining (*P <*0.01, **Figure S5E**). We further analyzed the macrophage phenotype and its function. The increase of CD86^+^ M1-type population (*P <*0.01) and mRNA level of M1 function markers (IL-12, IL-6, MHC-II, IFN-γ and TNF-α; *P <*0.01) and the decrease of CD209^+^/CD163^+^ M2-type population (*P <*0.05) and mRNA level of M2 function marker (IL-10; *P <*0.01) were also observed in the combination treatment groups but not in RT-alone group (**Figure 6C**). Such effects are not as strong as in the early stage of the treatment (**Figure 2B**). The increased of CD8^+^ T cell population (**Figure 6D**) with higher expression of granzyme-B (**Figure 6E**) and a reduction in vascular (**Figure 6F**), under the combination treatment were also observed, supporting the hypothesis that VPA/HPTA prolonged the RT effect by maintaining anti-tumor immune response through the later stage of treatment.

### VPA/HPTA Can Directly Promote M1 Polarization of Macrophages to Activate Anti-Tumor Response of CD8^+^ T Cells *In Vitro*

**Figure 7:**
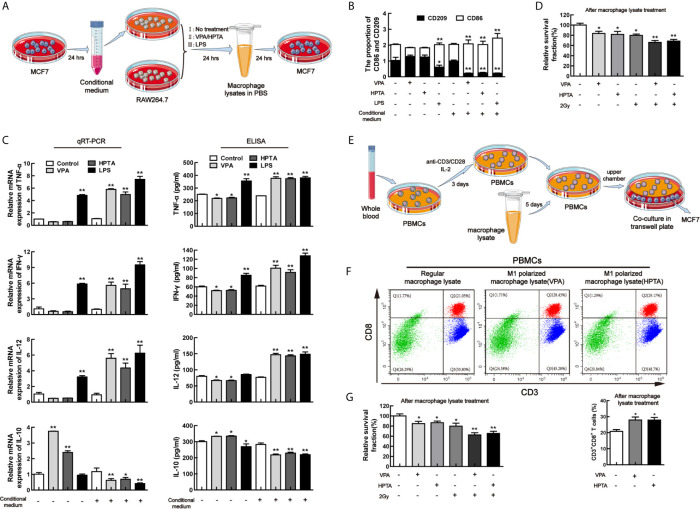
VPA/HPTA can directly promote M1 polarization of macrophages to activate anti-tumor response of CD8+ T cells *in vitro* **(A)** Protocol for MCF7 conditional medium and macrophage polarization experiment. **(B)** qRT-PCR analysis of markers (CD86 and CD209) of reprogrammed RAW264.7 macrophages under different treatment conditions. **(C)** qRT-PCR and ELISA analysis of cytokines (TNF-α, IFN-γ, IL-10 and IL-12) of reprogrammed RAW264.7 macrophages under different treatment conditions. **(D)** The survival of MCF7 cells treated with macrophage lysate was detected by MTT assay. **(E)** Protocol for extraction and activation of PBMCs and the co-culture with MCF7 cells. **(F)** Flow cytometric analysis of the effect of macrophage lysates on CD8^+^ T lymphocytes. **(G)** MTT results of survival of MCF7 cells after co-culture. Each data point in the graphs was from three independent experiments (mean ± SD). P-values were calculated by Student’s t-test (*P < 0.05, **P < 0.01).

To verify VPA-like compounds can directly reprogram M1 polarization and activate anti-tumor response, the conditional medium experiment was employed for this study. Firstly, to manipulate the environment for tumor cell growth, the conditional medium, which was from the culturing breast cancer cell line MCF7, was used to incubate the macrophage cells, RAW264.7, thus to investigate the effect of VPA/HPTA on RAW264.7 polarization. The experiment design was shown in **Figure 7A**. As a negative control, regular medium was used. Both the qRT-PCR and ELISA experiments demonstrated a significant decreased in the level of the M1 marker CD86 and its secreted cytokines (IL-12, IFN-γ, and TNF-α), and a significant increase in M2 secreted cytokine IL-10 were observed after VPA/HPTA treatment (*P <*0.01), although the significant changes in the level of the M2 marker CD209 were not observed (**Figures 7B, C**). The results indicate that VPA/HPTA can induce M2 polarization of macrophages under a normal culture environment.

With the conditional medium, through both qRT-PCR and ELISA, a significant elevation of the level of CD86 and the cytokines (IL-12, IFN-γ, and TNF-α) (*P <*0.01) and a significant decrease of the level of CD209 and IL-10 were found after VPA/HPTA treatment (*P <*0.01). The results indicate that VPA/HPTA can directly promote M1 polarization under tumor cell growth environment (**Figures 7B, C**).

We also used LPS as a positive control for this study. The results indicated that LPS can induce M1 polarization of macrophages under regular medium and conditional medium (**Figures 7B, C**), consistent with other reports (54, 55), suggesting that our experimental design was reliable.

Cell lysate from the macrophages was further used to incubate MCF7 cells to test for cell viability (**Figure 7D**). We found that the relative survival fraction of VPA/HPTA-alone was comparable to RT-alone treatment. The combination treatment resulted in further inhibited cell growth (*P <*0.01).

We concluded from the above results that VPA/HPTA can directly induce macrophage M1 polarization in the tumor environment, and activate macrophage-mediated anti-tumor immunity for enhancing the effects of radiotherapy to tumor.

Since VPA/HPTA can directly induce M1 polarization and result in the increase of IL-12 level, we next test the effect of VPA/HPTA-induced M1 polarization on CD8^+^ T cells *in vitro*. The cell lysate from the VPA/HPTA-treated macrophage RAW264.7 was used to treat isolated mononuclear cells extracted from venous blood from healthy donors, at the same time the isolated mononuclear cells were activated with anti-CD3/CD28. The experimental design was shown in **Figure 7E**. After treatment for 5 days, the mononuclear cells were labeled with the antibodies of CD3 and CD8 for isolating CD8^+^ T cells by flow analysis. The results showed that VPA/HPTA significantly increased the number of CD3^+^CD8^+^ T lymphocytes (**Figure 7F**, *P <*0.05), indicating that VPA/HPTA-induced M1 polarization can promote the proliferation of CD8^+^ T cells. Next, to further illustrate the effect of activated CD8^+^ T cells on the growth of tumor cell MCF7 (**Figure 7G**), the PBMCs treated by VPA/HPTA-treated macrophage lysate were co-cultured with MCF7 cells for 48 h. We found the viability of MCF7 cells was inhibited by VPA/HPTA-alone and RT-alone treatment (*P <*0.05), this was further reduced in the combination treatment groups (*P <*0.01). The results suggested that VPA/HPTA not only can activate macrophage-mediated anti-tumor immunity but also can activate macrophage-CD8^+^ T cell-mediated anti-tumor immunity to enhance the effects of RT to tumor, thus supported the earlier *in vivo* results.

## Discussion

**Figure 8:**
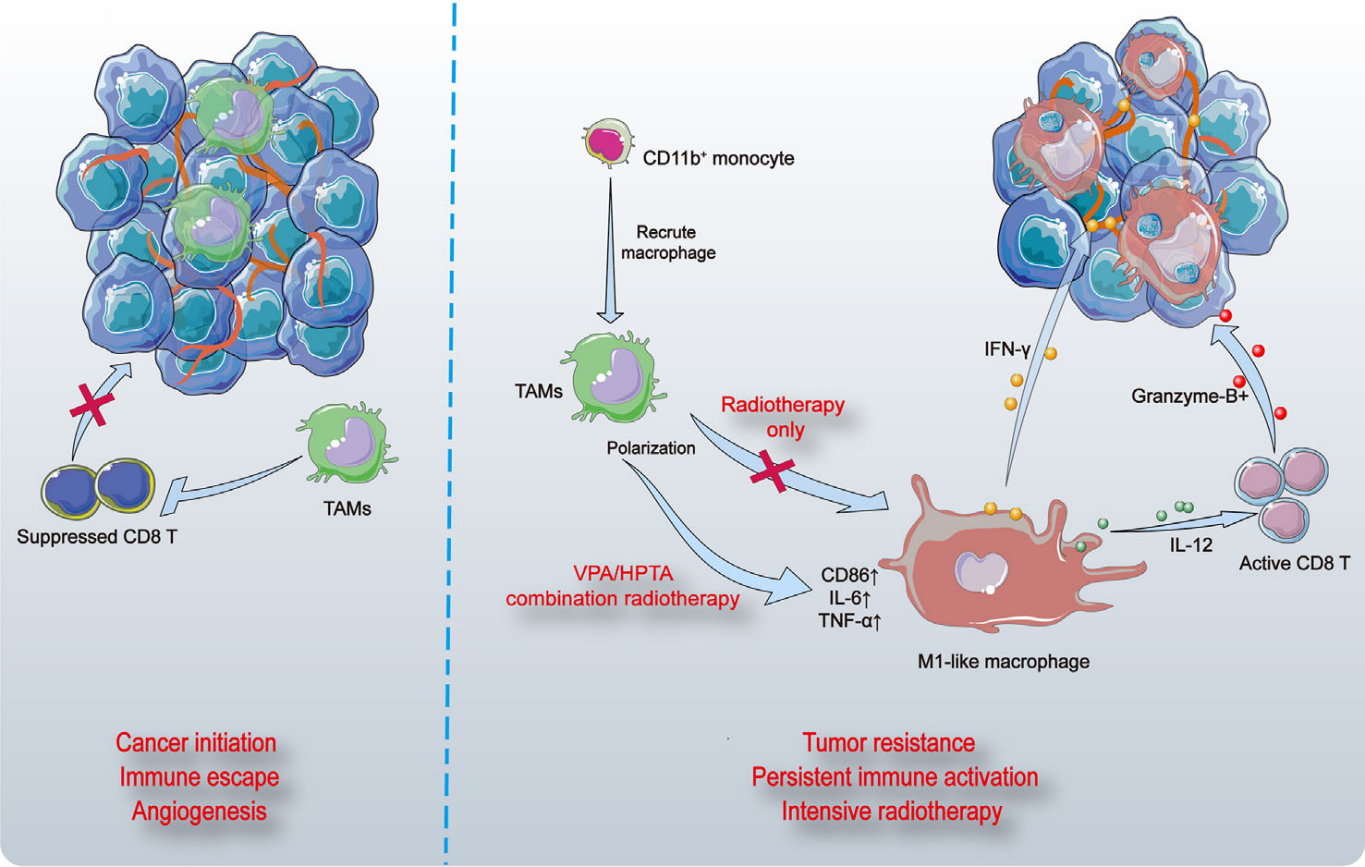
The model of VPA/HPTA to enhance and prolong the radiotherapy effect by activating and maintaining anti-tumor immune response. Breast tumors in rats induced by DMBA contain abundant vasculature and pro-tumor macrophages (TAMs) that suppress the function of CD8+ T cells (left). Myeloid-derived cells are recruited to tumor sites, differentiate into macrophages, and further polarize toward M1 phenotype, thus promote inflammatory response. CD8+ T cells are activated, granzyme-B is secreted possibly through the IL-12 pathway, thereby killing tumors. The vasculature of tumors becomes sparse, possibly due to stimulation by IFN-γ. At the same time, the combination treatment not only effectively improve the effect of radiotherapy during the immediate exposure, the concurrent therapy also delay the growth of tumors and prolong the anti-tumor effect by continuously activating the immune response to compensate for recurrence after radiotherapy.

In this study, we demonstrate that VPA/HPTA evokes immune activation by mobilizing myeloid-derived macrophages and triggering M1 polarization in a DMBA-induced rat breast cancer model. These reprogrammed macrophages led to subsequent T cell recruitment and activation, vascular normalization, and tumor suppression (**Figure 8**). Our findings support the proposition of VPA/HPTA as an adjuvant therapy to low-dose radiotherapy in breast cancer; VPA/HPTA enhances and prolongs the RT effect on breast cancer by activating and maintaining the anti-tumor immune function.

### Persistent Immune Activation Is the Key to Prevent Tumor Recurrence

RT has been the mainstay of oncological treatment of breast cancer since the 1900s; today, about 50–60% of cancer patients continue to receive this treatment modality. However, the resistance of tumor cells to RT and high cancer recurrence rate has been reported (56). Understanding the mechanism of radiation resistance in breast cancer is of clinical importance. The tumor microenvironment has been known to influence the response to RT, specifically lymphocytes, monocytes and macrophages are particularly radiosensitive. Furthermore, ionizing radiation has an effect on the vascular endothelium and affects the recruitment of anti-tumor T cells into the tumor site, as well as initiating adaptive and innate immune responses that can result in systemic anti-tumorigenic effects both inside and outside of the irradiation field.

Studies have shown that cancer immunotherapy achieves a durable clinical response in patients with advanced cancer, who are refractory to conventional treatment (57). While RT can also activate the immune system to some extent (58), it is limited by the dose and frequency of RT. Such RT-induced immune activation is short-lived and tumors are prone to recurrence. Therefore, safer and more effective immune activators are needed to supplement and complement RT.

### VPA-Like Compounds Are Ideal Immune Activators, Which Can Activate the Anti-Tumor Response of CD8^+^ T Cells and Enhance and Prolong the Curative Effect of Radiotherapy

The microenvironment plays an important role in the progress of breast cancer and its resistance to treatment (36). Most solid tumor microenvironments tend to have a certain amount of TAMs and are associated with tumor invasion and poor prognosis (17–19, 59). TAMs were found to enhance malignancy by stimulating angiogenesis, inducing tumor cell migration, invasion and infiltration, and inhibiting anti-tumor immunity in mouse models (60). In our working model, VPA/HPTA induces an increase in myeloid-derived macrophages and activates polarization toward a M1 phenotype that is pro-inflammatory and has phagocytic capacity.

Analysis of breast cancer patients indicates that a low ratio of macrophages to CD8^+^ T cells is associated with poorer survival, suggesting that macrophages may play a major role in suppressing T cell activity against tumors (61). CD8^+^ T cells play a key role in anti-tumor immunity, but their activity is inhibited in the tumor microenvironment, therefore tumors can escape immune attack by various mechanisms of immunosuppression (62–65). The cytotoxicity of reactivated CD8^+^ T cells has important clinical significance in cancer immunotherapy. Here, we explored a novel combination treatment modality that activates the anti-tumor CD8^+^ T cells through regulation of the tumor microenvironment to enhance the efficacy of RT. We demonstrate that VPA/HPTA can reprogram macrophages in tumors, activate CD8^+^ T cell-mediated anti-tumor immune response, and enhance radiotherapy efficacy.

### Additional Implementation of Immune Checkpoint Inhibitors May Have a Further Positive Impact on the Treatment Efficacy in Our Model

Immune checkpoints are immunosuppressive pathways that maintain self-tolerance and protect surrounding tissues by modulating immune responses, a property that tumor cells exploit to evade attack by immune cells. Currently, two of the most extensively studied immune checkpoint targets in tumors are Cytotoxic T lymphocyte associated antigen 4 (CTLA-4) and the PD-1 receptor. Immune checkpoint inhibitors release the “immune brakes” in the tumor microenvironment, reactivate the immune response effect of T cells on tumors, thereby achieving anti-tumor effects. It is also of interest whether immune checkpoint inhibition and other immunotherapies can be combined to better exert anti-tumor effects.

Studies have reported that the triple combination of anti-CTLA-4, anti-PD-1, and G47Δ-mIL12 was able to cure most mice of glioma (66, 67). This treatment was associated with macrophage influx and M1-like polarization, along with increased T effector to T regulatory cell ratios. Among them, G47Δ-mIL12 induces M1-like polarization in TAMs. This synergy may permit low dose of the immune checkpoint inhibitors to reduce potential adverse effects (67). In our study, VPA/HPTA seems to act similarly to G47Δ-mIL12 by mobilizing macrophages to recruit and trigger M1 polarization, suggesting that administration of immune checkpoint inhibition (anti-CTLA-4, anti-PD-1) in our model may potentially achieve better therapeutic outcomes. Furthermore, VPA has been used clinically for decades and is a low-cost alternative to the currently available immune checkpoint inhibitor such as ipilimumab, pembrolizumab and nivolumab, especially so for resource-constraint countries.

It was previously shown that the combination of RT, anti-CTLA4, and anti-PD-L1 promotes immunity through distinct mechanisms. Anti-CTLA4 predominantly inhibits T regulatory cells (Tregs) to increase the CD8 T cell to Treg (CD8/Treg) ratio. RT enhances the diversity of the T cell receptor (TCR) repertoire of intratumoral T cells. Together, anti-CTLA4 promotes expansion of T cells, while RT shapes the TCR repertoire of the expanded peripheral clones. PD-L1 blockade reverses T cell exhaustion and attenuates the decrease in the CD8/Treg ratio, further encourages oligo-clonal T cell expansion (68). This suggests that the combination of RT with immune checkpoint inhibitor can improve tumor immunotherapy efficacy. Thus, we speculate that the addition of immune checkpoint inhibitor to existing treatment modalities may have a further positive impact on treatment efficacy.

### The Specific Immune Activation Mechanism and More Reasonable Strategies of VPA-Like Substances Need to Be Further Explored

We found that CD11b^+^ cells infiltrate tumors, but did not determine which stimuli and receptors were involved in this recruitment. There are several possibilities for the exact source of recruitment of CD11b^+^ cells and we cannot completely exclude the presence of CD11b^+^ MDSCs (Myeloid-derived suppressor cells). However, MDSCs, as immunosuppressive cells, induce the generation of Tregs (Regulatory cells) (69), promote the transformation of macrophages from M1 to M2 phenotype (70), thus leading to increased TAMs differentiation and vascular endothelial cells (71) as well as inhibiting the killing of tumor cells by T cells (72) to achieve anti-tumor immunosuppression. In our study, TAMs were polarized from M2 phenotype to M1 with VPA/HPTA-alone treatment as well as in combination with RT. Meanwhile, CD8^+^ T cells were induced to secrete granzyme B to restrain tumor, and CD31 immunofluorescence staining also indicated that the tumor vessels became sparse. These findings all confirmed that the recruited CD11b^+^ cells were not MDSCs; if any, minimal. Study has reported additional roles for CD11b (45): CD11b activation promotes pro-inflammatory macrophage polarization by stimulating the expression of microRNA *Let7a*. In contrast, inhibition of CD11b prevents *Let7a* expression and induces cMyc expression, leading to immune suppressive macrophage polarization, vascular maturation, and accelerated tumor growth. This suggests that CD11b may serve as a positive regulator of immune activation and a target for cancer immunotherapy.

At the same time, we also found that although the growth of tumor volume was inhibited after the combination treatment as compared with radiotherapy alone, the tumor nonetheless continued to grow abide at a much slower growth rate, suggesting that rebound effect may nonetheless occur after stopping combination treatment (73). If we are to extend the duration of VPA/HPTA treatment, the stability of reprogramming phenotype and toxicology would warrant further exploration.

The strikingly different effects of VPA/HPTA on macrophage polarization demonstrated in the cell model *in vitro*, with and without the tumor cell medium environment, allow us to make bold speculation that in the animal model, in addition to promoting M1 polarization of macrophages to activate anti-tumor response of CD8^+^ T cells, VPA/HPTA may also exhibit protection against the injury of distant normal tissues induced by RT, as it is possible to mediate anti-inflammatory effects *via* macrophage M2 type polarization.

As for how CD8^+^ T cells may kill the tumor cells, the perforin/granzyme-B apoptosis pathway is a likely candidate (74), but there are also reports that T cell-promoted tumor ferroptosis is an anti-tumor mechanism (75), which needs further exploration.

Regardless of these hitherto untested possibilities, VPA/HPTA interventions are safe and effective options for the treatment of breast cancer: persistent immune activation and intensive radiotherapy. Our study may provide a more rational and long-term strategy for breast cancer treatment in clinic.

## Data Availability Statement

The original contributions presented in the study are included in the article/**Supplementary Material**. Further inquiries can be directed to the corresponding author.

## Ethics Statement

The studies involving human participants were reviewed and approved by Shandong University Human and Animal Ethics Research Committee (81472800, approved March 2014). The patients/participants provided their written informed consent to participate in this study. The animal study was reviewed and approved by Shandong University Human and Animal Ethics Research Committee (81472800, approved March 2014).

## Author Contributions

ZC, DL and ZF designed the study, analyzed the data, and wrote the manuscript. ZC performed most of the experiments. GL, CC, LJ, WD, CDi, QS and JP finished the rest part of the experiments in this study, and they analyzed the data and designed the figures. CDo and FZ provided guidance for this work. All authors provided critical feedback on the manuscript. All authors contributed to the article and approved the submitted version.

## Funding

This research was supported by grants from National Natural Science Foundation of China (No. 81472800), and Department of Science and Technology of Shandong Provence (2019GSF108083 and ZR2020MH330).

## Conflict of Interest

The authors declare that the research was conducted in the absence of any commercial or financial relationships that could be construed as a potential conflict of interest.

## Abbreviations

VPA, valproic acid; HPTA, 2-hexyl-4-pentynoic acid; DAB, 3,3'-diaminobenzidine; DMBA, 7,12-dimethylbenz[a]anthracene; FBS, fetal bovine serum; HDACi, histone deacetylase inhibitor; BrdU, 5-Bromo-2′-deoxyuridine; HE, hematoxylin and eosin; IHC, immunohistochemistry; SD, Sprague–Dawley; TBS, Tris-Buffered Saline; TAMs, tumor-associated macrophages; CTLs, cytotoxic T lymphocytes; PD1, programmed cell death protein 1; EMT, Epithelial–mesenchymal transition; PBMCs, Peripheral blood mononuclear cells; MDSCs, Myeloid-derived suppressor cells; Tregs, Regulatory cells; CTLA-4, Cytotoxic T-lymphocyte-associated protein 4.
