# Supplementary material for: Valproic Acid-Like Compounds Enhance and Prolong the Radiotherapy Effect on Breast Cancer by Activating and Maintaining Anti-Tumor Immune Function
Source: Front Immunol. 2021 May 12;12:646384. doi: 10.3389/fimmu.2021.646384 (PMC8149798; doi:10.3389/fimmu.2021.646384)
Supplement: Supplementary file 1 [file DataSheet_1.docx]

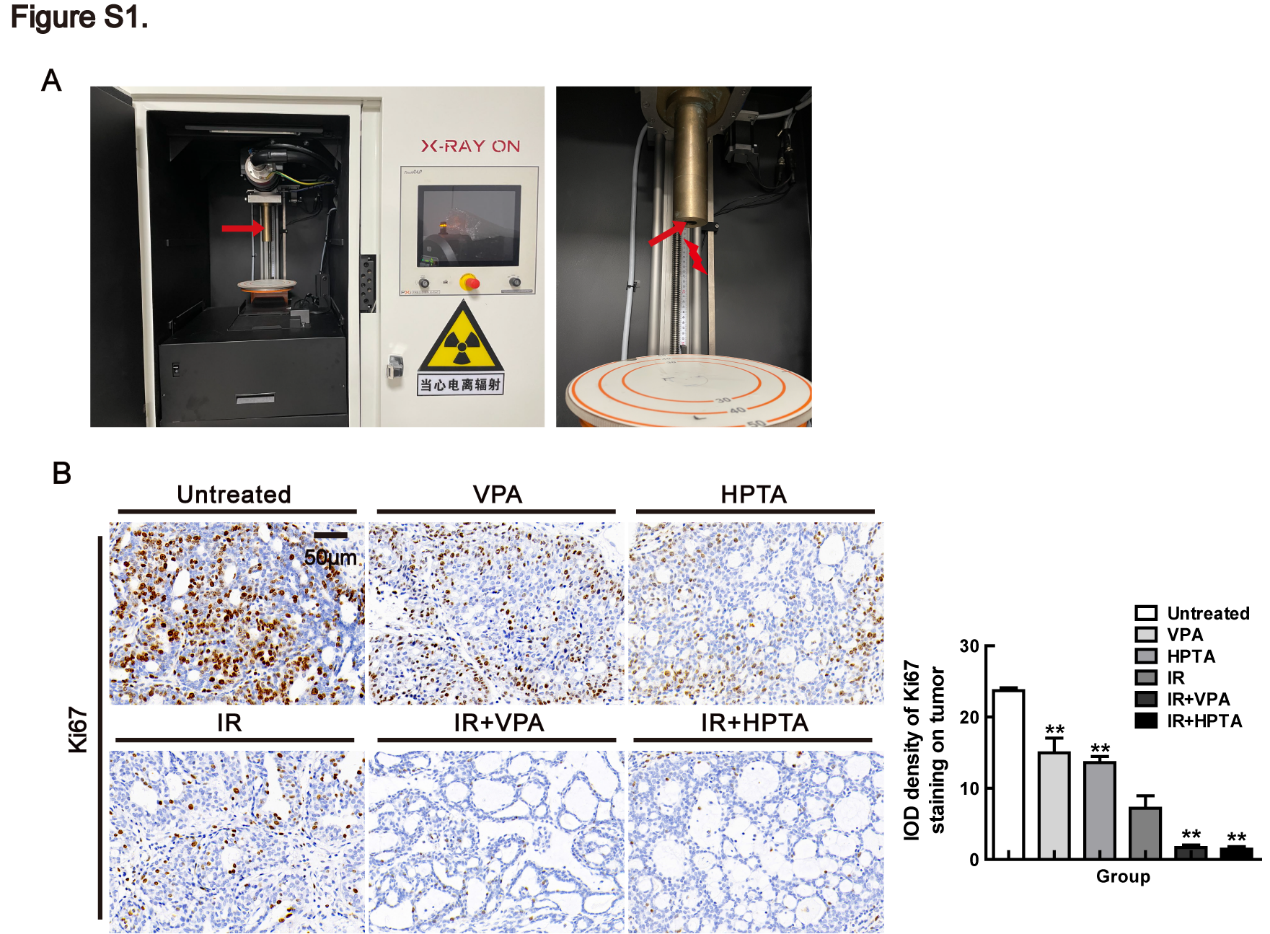


Figure S1. VPA/HPTA enhanced radiotherapy effect to inhibit tumor growth in rats with breast cancer

(A) X-ray Irradiator, the apparatus used for the radiotherapy of tumors in the experiment. (B) IHC was performed on tumor sections for a marker of proliferation, Ki67. Quantitation as a percentage of total tissue is shown to the right of representative images. Each data point in the graphs was from three independent experiments (mean ± SD). *P*-values were calculated by Student’s t-test (* *P*<0.05, ** *P*<0.01).


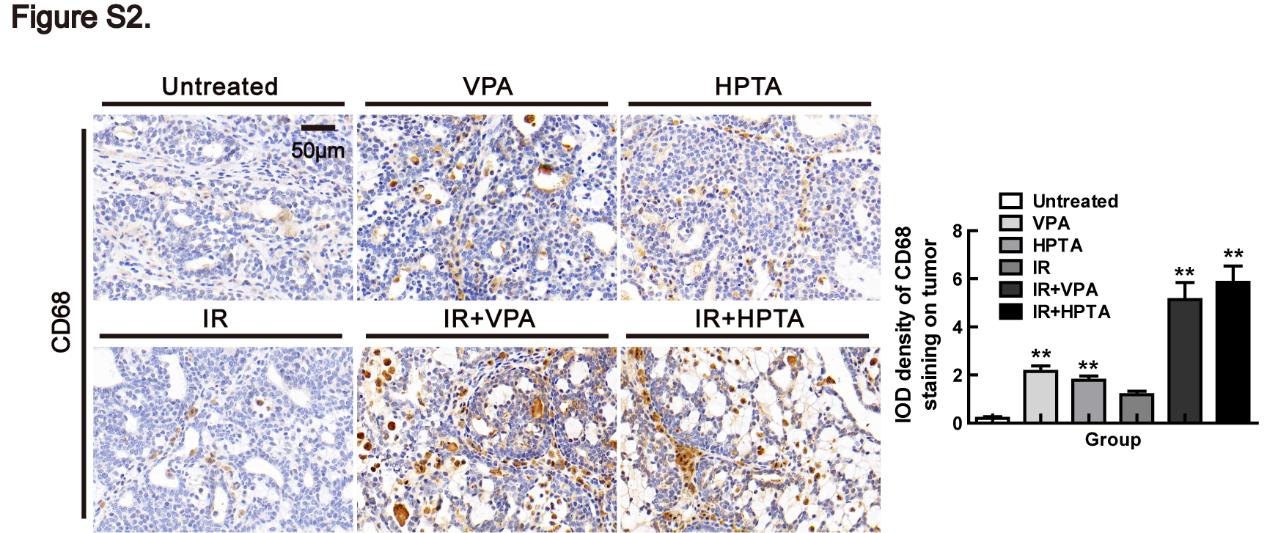


Figure S2. VPA/HPTA activates the macrophages and reprograms TAMs polarization towards M1 phenotype in irradiated breast tumor at the early stage of the treatment

IHC was performed on tumor sections for and the macrophage-specific marker CD68 to assess infiltration of macrophages, and representative quantitation and images are shown. Each data point in the graphs was from three independent experiments (mean ± SD). *P*-values were calculated by Student’s t-test (* *P*<0.05, ** *P*<0.01).


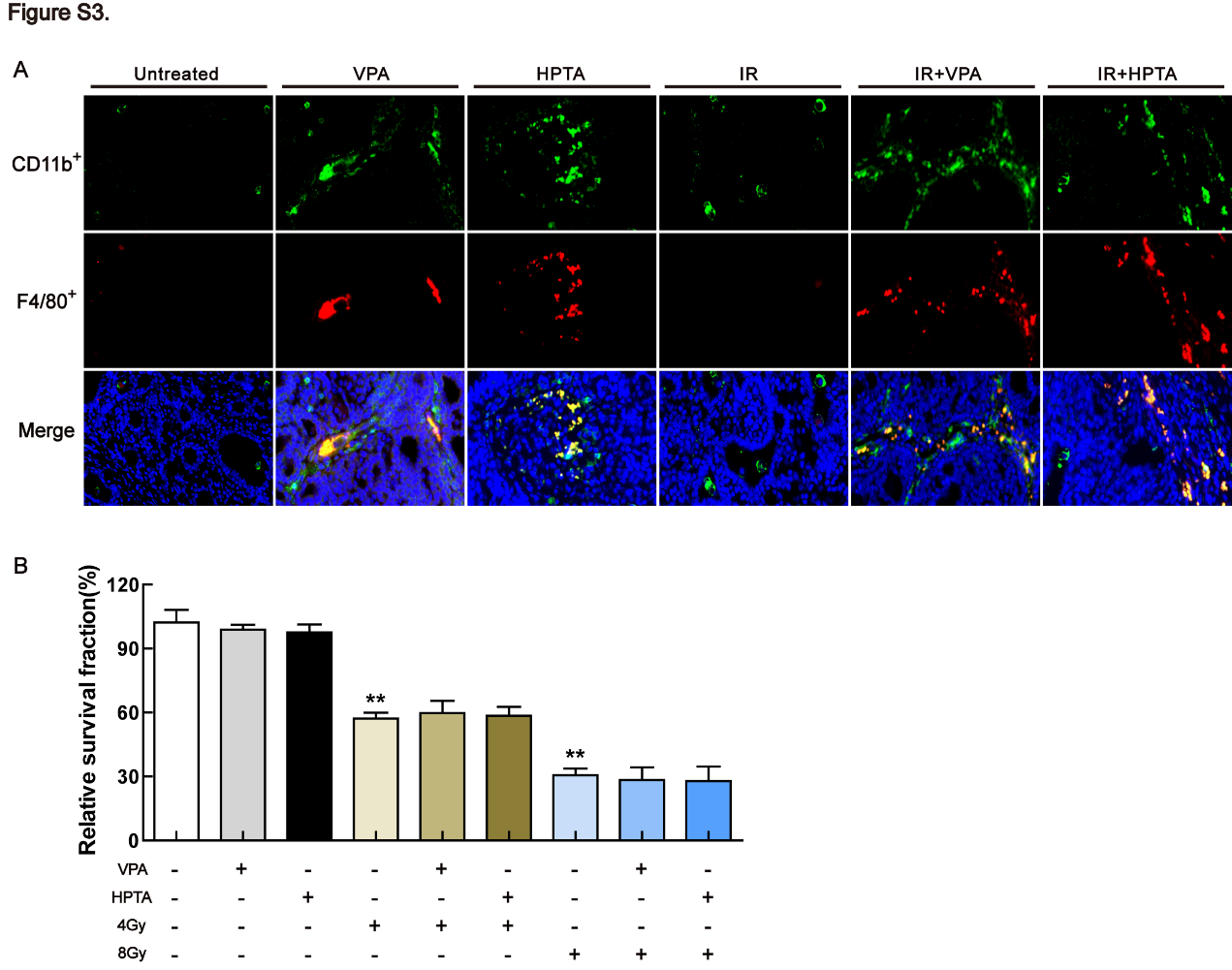


Figure S3. VPA/HPTA regulates myeloid-derived macrophages to enhance the radiotherapy effect in breast cancer at the early stage of treatment in vivo

(A) Immunofluorescence co-staining of myeloid-derived cells (CD11b^+^: green) and macrophages (F4/80^+^: red) (B) The ability of RAW264.7 macrophages was tested after IR (4 Gy and 8 Gy) and VPA/HPTA combination treatment by MTT assay in vitro.


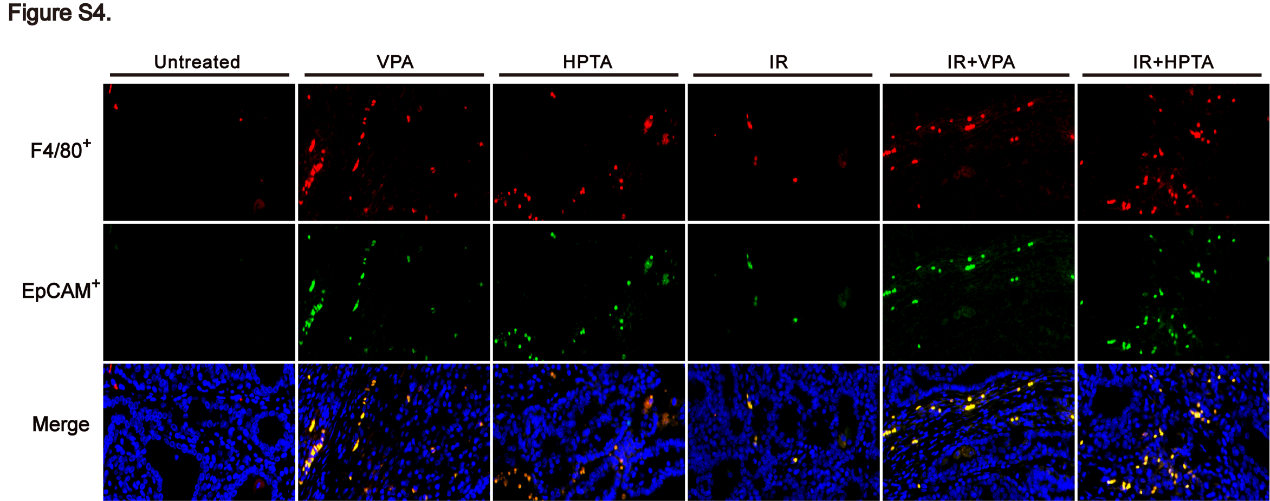


Figure S4. VPA/HPTA-activated macrophages are highly phagocytic in breast tumors at the early stage of treatment in vivo

Phagocytosis of breast tumor cells was quantified as the proportion of F4/80^+^ (red) macrophages that contain intracellular EpCAM (green), a marker of breast tumor cells, by immunofluorescence.

**
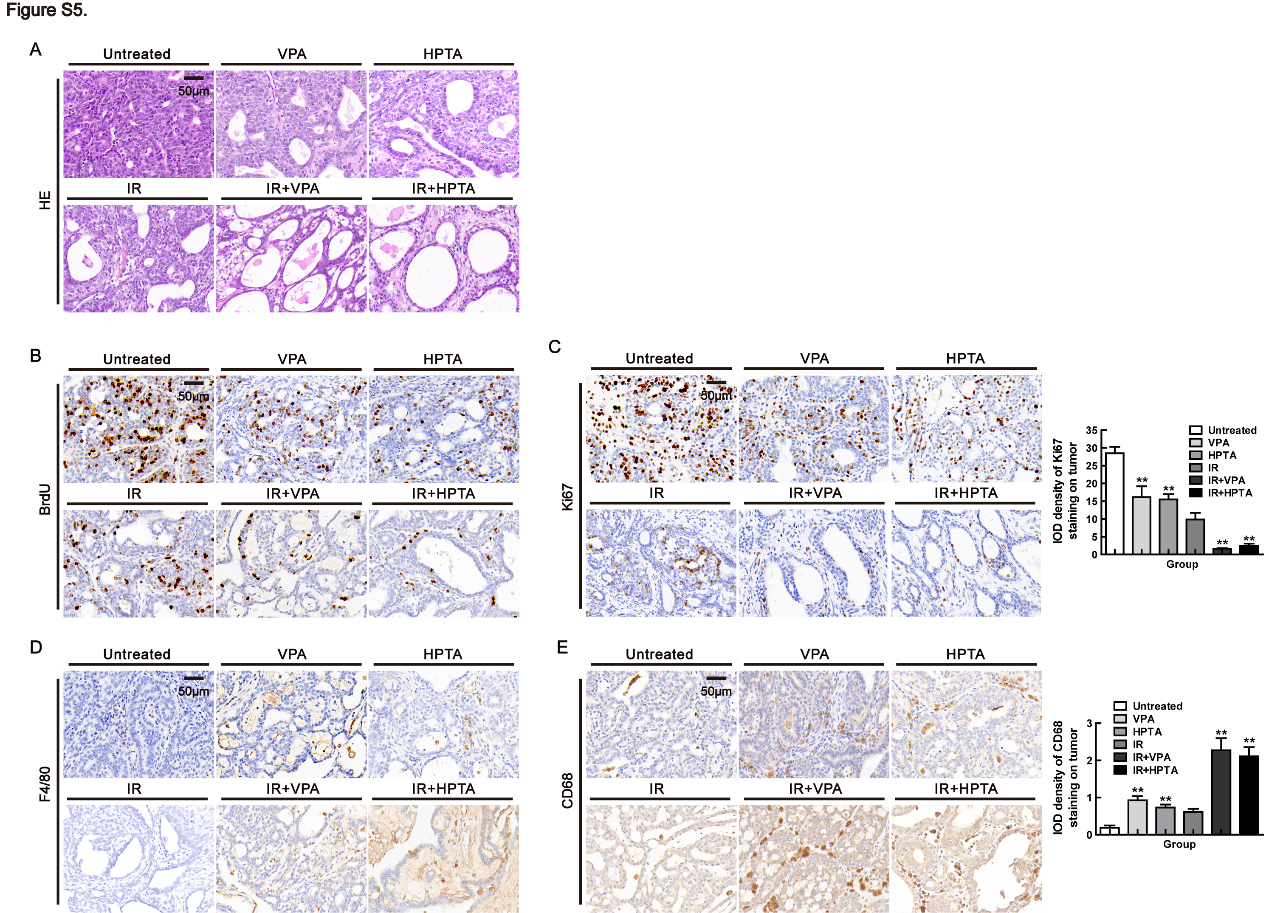
**

Figure S5. VPA/HPTA prolong the radiotherapy effect of breast cancer via maintaining the durability of anti-tumor immune response in vivo

Tumor tissues were analyzed 70 days after treatment. (A) HE staining for the morphology of tumors in different groups. (B) Representative images of BrdU staining. (C) IHC for a marker of proliferation, Ki67. Quantitation as a percentage of total tissue is shown to the right of representative images. (D) Representative images of F4/80 staining. (E) IHC for the macrophage-specific marker CD68 to assess infiltration of macrophages, representative quantitation and images are shown. Each data point in the graphs was from three independent experiments (mean ± SD). *P*-values were calculated by Student’s t-test (* *P*<0.05, ** *P*<0.01).
