# Supplementary material for: Valproic Acid-Like Compounds Enhance and Prolong the Radiotherapy Effect on Breast Cancer by Activating and Maintaining Anti-Tumor Immune Function
Source: Front Immunol. 2021 May 12;12:646384. doi: 10.3389/fimmu.2021.646384 (PMC8149798; doi:10.3389/fimmu.2021.646384)
Supplement: Supplementary file 2 [file Table_1.docx]

**Table 1**

**The primer sequences used in this study**

|  | **Oligo Name** | **Primer Sequence (5’-3’)** |
| --- | --- | --- |
| **Rat** | ***CD86*** | **Forward: AAG CCC GTG TCC TTG ATC TG** |
|  |  | **Reverse: AGA CAT GTG TAA CCT GCA CCA T** |
|  | ***CD209*** | **Forward: CAC CTC TGT CAC CCA GCT TT** |
|  |  | **Reverse: CGG GTA TCG CAT CTG AGG TC** |
|  | ***IL-10*** | **Forward: CTG CTC TTA CTG GCT GGA GTG AAG** |
|  |  | **Reverse: TGG GTC TGG CTG ACT GGG AAG** |
|  | ***TNF-α*** | **Forward: ATG GGC TCC CTC TCA TCA GTT CC** |
|  |  | **Reverse: GCT CCT CCG CTT GGT GGT TTG** |
|  | ***IL-6*** | **Forward: ACT TCC AGC CAG TTG CCT TCT TG** |
|  |  | **Reverse: TGG TCT GTT GTG GGT GGT ATC CTC** |
|  | ***IL-12*** | **Forward: CCT CAA GTT CTT CGT CCG CAT CC** |
|  |  | **Reverse: CAT TGG ACT TCG GCA GAG GTC TTC** |
|  | ***CD163*** | **Forward: AGC ATG GCA CAG GTC ATT CA** |
|  |  | **Reverse: GGT CAC AAA ACT TCA ACC GGA** |
|  | ***MHC-Ⅱ*** | **Forward: TGT GGT TGT GCT GAT GGT GCT G** |
|  |  | **Reverse: GCT GCG TCC CGT TGG TGT AG** |
|  | ***IFN-γ*** | **Forward: ACA ACC CAC AGA TCC AGC ACA AAG** |
|  |  | **Reverse: CAC CGA CTC CTT TTC CGC TTC C** |
|  | ***GAPDH*** | **Forward: AGT GCC AGC CTC GTC TCA TA** |
|  |  | **Reverse: GAT GGT GAT GGG TTT CCC GT** |
| **Mouse** | ***CD86*** | **Forward: ACG GAG TCA ATG AAG ATT TCC T** |
|  |  | **Reverse: GAT TCG GCT TCT TGT GAC ATA C** |
|  | ***CD209*** | **Forward: CAG TTG AAG GCT GGC GTA GAT CG** |
|  |  | **Reverse: GTG GCA GGC AGT GGC AGA ATC** |
|  | ***IL-10*** | **Forward: TTC TTT CAA ACA AAG GAC CAG C** |
|  |  | **Reverse: GCA ACC CAA GTA ACC CTT AAA G** |
|  | ***TNF-α*** | **Forward: ATG TCT CAG CCT CTT CTC ATT C** |
|  |  | **Reverse: GCT CCT CCG CTT GGT GGT TTG** |
|  | ***IL-12*** | **Forward: TGA GAA GTA TTC AGT GTC CTG C** |
|  |  | **Reverse: CTG TGA GTT CTT CAA AGG CTT C** |
|  | ***IFN-γ*** | **Forward: CTT GAA AGA CAA TCA GGC CAT C** |
|  |  | **Reverse: CTT GGC AAT ACT CAT GAA TGC A** |
|  | ***GAPDH*** | **Forward: GGT TGT CTC CTG CGA CTT CA** |
|  |  | **Reverse: GGT GGT CCA GGG TTT CTT ACT C** |
